# Circulating Tumor Cell Analysis in Preclinical Mouse Models of Metastasis

**DOI:** 10.3390/diagnostics8020030

**Published:** 2018-04-28

**Authors:** Jenna Kitz, Lori E. Lowes, David Goodale, Alison L. Allan

**Affiliations:** 1 London Regional Cancer Program, London Health Sciences Centre, Department of Anatomy & Cell Biology, Western University, London, ON N6A 5W9, Canada; jkitz@uwo.ca; 2 Flow Cytometry and Special Hematology, London Health Sciences Centre, London, ON N6A 5W9, Canada; lori.lowes@lhsc.on.ca; 3 London Regional Cancer Program, London Health Sciences Centre, London, ON N6A 5W9, Canada; david.goodale@lhsc.on.ca; 4 London Regional Cancer Program, London Health Sciences Centre, Departments of Anatomy & Cell Biology and Oncology, Lawson Health Research Institute, Western University, London, ON N6A 5W9, Canada

**Keywords:** circulating tumor cells, preclinical models, metastasis

## Abstract

The majority of cancer deaths occur because of metastasis since current therapies are largely non-curative in the metastatic setting. The use of in vivo preclinical mouse models for assessing metastasis is, therefore, critical for developing effective new cancer biomarkers and therapies. Although a number of quantitative tools have been previously developed to study in vivo metastasis, the detection and quantification of rare metastatic events has remained challenging. This review will discuss the use of circulating tumor cell (CTC) analysis as an effective means of tracking and characterizing metastatic disease progression in preclinical mouse models of breast and prostate cancer and the resulting lessons learned about CTC and metastasis biology. We will also discuss how the use of clinically-relevant CTC technologies such as the CellSearch^®^ and Parsortix™ platforms for preclinical CTC studies can serve to enhance the study of cancer biology, new biomarkers, and novel therapies from the bench to the bedside.

## 1. Introduction

Cancer is currently one of the leading causes of death worldwide with the projected number of cases expected to increase 50% from 2012 to 2030 [1]. The five-year survival rates for non-metastatic prostate and breast cancers in the United States range from 93% to 100% [2,3]. However, as these cancers progress, cells may escape from the primary tumor and spread to distant organs in the body, through a deadly process called metastasis [4]. Approximately 90% of cancer-related deaths occur as a result of metastasis [5]. Correspondingly, five-year survival rates for metastatic prostate and breast cancer drop to approximately 29% and 22%, respectively [2,3]. Recurrence is also seen in 30% to 40% of patients after successful treatment of a primary tumor, of which 50% is a recurrence of metastatic disease [4,6].

Current therapies are non-curative towards these aggressive cancers. Therefore, more research is required to successfully identify and treat patients with metastatic cancer. In particular, the use of in vivo preclinical animal models of metastasis provide an important opportunity to study and develop novel biomarkers such as circulating tumor cells (CTCs) in controlled experimental studies, which, in turn, may also provide information for developing new clinical treatments targeted against metastatic disease. This review will provide a brief overview of the metastatic process and how it is detected clinically including the use of imaging and CTC assays. We will then highlight different approaches for modeling and tracking metastatic progression in vivo in the preclinical setting with a particular focus on CTC generation and CTC assays that can be implemented for the preclinical development and/or optimization of new metastasis biomarkers.

## 2. The Metastatic Process

**Figure 1:**
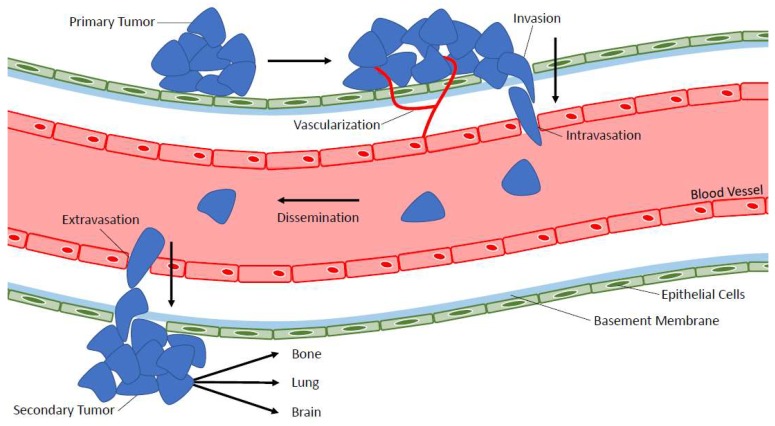
The metastatic process. The multistep process of metastasis is complex and includes primary tumor vascularization, escape/intravasation of tumor cells into the bloodstream, survival in the bloodstream, and dissemination throughout the body, extravasation out of the bloodstream, and secondary tumor formation in distant organs.

Metastasis is the spread of cancer from the primary tumor to a secondary location within the body [7] and different types of cancer have specific affinities for certain target organs, which support growth of secondary tumors [8]. The multi-step process of metastasis is complex (see Figure 1) and begins with the local infiltration of cancer cells out of the primary tumor into the surrounding vasculature [9]. Angiogenesis, the growth of a vascular network formed from pre-existing vessels, is an important step in facilitating tumor cell escape from the primary tumor [9,10]. Once in the bloodstream, cancer cells must survive physical shear stress and/or immune challenge in the circulation in order to disseminate throughout the body [9]. These cells often arrest in capillary beds of distant organs and can then extravasate out of the bloodstream into the secondary organ. Here they may die, remain dormant, or proliferate in order to colonize a metastatic tumor at a secondary location [9]. According to the seed and soil hypothesis [8], each cancer type has a specific pattern of metastasis called organ tropism. For example, when considering the four most common types of cancer [11], lung cancer will metastasize to the brain, bones, and adrenal glands [12] while breast cancer will metastasize to bones, lungs, the liver, and the brain [13]. Prostate cancer metastasizes to bones, lungs, the liver, the brain, and lymph nodes [14,15] and colorectal cancer metastasizes to the lungs, the liver, and the peritoneum [16].

## 3. Clinical Imaging Techniques for Identifying and Tracking Metastasis

In order to develop therapies aimed at treating metastasis, clinical imaging technologies must be able to sensitively detect and monitor metastasis. While there are a wide range of approaches available to assess disease progression in patients, only a few emerge as reliable and commonly performed. Each have their own advantages and disadvantages.

### 3.1. Whole Body-Bone Scanning

Detection of bone metastases has traditionally been accomplished using whole body-bone scanning (WBS) or scintigraphy [6]. These scans are typically 15–20 min in duration during which a patient will lie inside the imaging instrument, which rotates around the patient transmitting radiation to take X-ray-like images [17]. WBS can be used with magnetic resonance imaging (MRI), positron emission tomography (PET), and/or computed tomography (CT) technologies to obtain the whole-body images (described further below) [18]. Studies have shown that up to 80% of prostate cancers, 70% of breast cancers, and 15% to 30% of lung, colon, stomach, bladder, uterus, rectum, thyroid, or kidney cancers will metastasize to the bone [19,20,21]. This metastasis imaging technique is mainly reserved for patients with high-risk cancers rather than for lower-risk cancer patients who are unlikely to have metastasis [22]. WBS provides detection of whole-body bone metastasis at low cost and is readily available [6]. However, WBS has low specificity for detecting bone metastasis with reported inadequate sensitivities in 70% to 90% of patients resulting in false-positive bone scans due to the inability of distinguishing between metastasis and other pathological conditions [23]. Consequently, WBS (as a sole imaging technique) is considered imprecise for diagnosing cancer metastasis [6].

### 3.2. Single Photon Emission Computed Tomography

Single photon emission computed tomography (SPECT) is another imaging technique commonly used to assess bone metastasis in cancer patients [24] as well as liver, lung, and lymph node metastasis [25,26]. SPECT takes two-dimensional images from single or multiple views to estimate three-dimensional radioactivity distribution inside a patient [22,27]. SPECT has higher diagnostic accuracy compared to WBS when used to detect bone malignancies and is able to be directly compared to other tomography-based techniques including the MRI [28]. However, SPECT has its own set of challenges with regards to image reconstruction [27]. The amount of radiopharmaceutical that can be administered is also limited by the maximum allowable dose of radiation to the patient, which results in a limited number of photons that can be used for imaging [27]. Collimator limitations can also decrease the photons available for imaging and can be associated with a loss of spatial resolution [27]. Lastly, since non-specific uptake in nonmalignant bone lesions may negatively impact the accuracy of bone scans using SPECT, it is common to use further examination techniques such as CT imaging or MRI in conjunction with SPECT when assessing a patient for metastatic disease [28].

### 3.3. Magnetic Resonance Imaging

Magnetic resonance imaging (MRI) uses a magnetic field to produce detailed images of cancer in patients [29]. These images can be used to assess localization for staging and to evaluate tumor aggressiveness along with providing guidance for patient treatment [30]. MRI uses non-radioactive contrast media and has better sensitivity and specificity for detecting metastasis compared to SPECT and WBS [23,31]. MRI is also commonly used to assess brain metastasis [32] as well as lesions in the liver and lymph nodes [33]. Unlike WBS and SPECT, MRI is increasingly performed in low-risk and intermediate-risk cancer patients as part of surveillance for metastasis and to determine eligibility for biopsies in less aggressive cancers such as prostate cancer [30]. However, in many cases, MRI can produce false-positive results by detecting non-specific bone lesions, which are of unknown clinical significance [30]. Importantly, because of the magnetic nature of MRI, patients who have metal implants in their body (such as cochlear implants, clips for brain aneurysms, coils in blood vessels, pacemakers, and more) are unable to have an MRI scan due to the possibility of damaging or dislodging the metal devices [34]. This limits the patient population and may exclude many patients who would benefit from MRI imaging to assess cancer metastasis.

### 3.4. Positron Emission Tomography and Computed Tomography

Positron emission tomography (PET) uses radiotracers to evaluate changes in organ and tissue functions at a cellular level [35]. PET has the ability to detect the onset of disease earlier than many other imaging techniques. However, multiple studies have reported such a large selection of PET tracers that it becomes difficult to designate which tracer is best since each has unique advantages and disadvantages [6,22,23,36]. For example, in prostate cancer patients, fluoro-d-glucose PET has been shown to lack sensitivity while fluoride PET lacks specificity. While prostate-specific membrane antigen-targeted PET shows promise, this tracer lacks evidence from clinical trials evaluating the role of clinical parameters such as androgen-sensitivity versus castrate-resistance [36]. Other available PET tracers include ^11^C-labeled or ^18^F-labeled choline and acetate, ^11^C-methionine, ^18^F-fluorodihydrotes-testosterone, and ^18^F-Fluoride [37]. However, an emerging theme in the extensive research comparing PET techniques is the combination of PET and computed tomography (CT) to enhance metastasis detection in patients [22,23,36]. PET/CT can take advantage of specific PET properties such as high sensitivity (depending on which tracer is used) and can reduce the risk of false-positives by using CT data to confirm the morphology of scintigraphic lesions [38]. PET/CT imaging can be used to assess a large variety of metastatic sites including lymph nodes, lungs, bones, and liver [39,40,41,42].

Despite advances in clinical imaging technologies, challenges remain for accurate detection and tracking of metastasis in cancer patients, particularly at earlier stages of the metastatic process. Available imaging approaches are often limited to detecting more advanced metastatic tumors because of issues with sensitivity and specificity, particularly if the location of metastasis is not known. The cost of instrumentation [43,44] and experienced clinical radiology personnel can also be a limitation particularly for smaller or remote hospitals. In the preclinical laboratory setting, imaging of metastasis in animal models is also challenged by the high costs associated with sophisticated imaging equipment including instruments for assessing fluorescence/luminescence as well as clinically-relevant imaging such as WBS, MRI, and PET/CT. In addition, it is also necessary for labs to employ experienced technicians to carry out the imaging and interpret the results. Therefore, large imaging equipment is often not a feasible option for preclinical laboratories with modest operating budgets. An emerging alternative approach which may help address these challenges in both the clinical and preclinical settings involves blood-based detection and tracking of metastatic disease including assays for analysis of circulating tumor cells (CTCs) [45].

## 4. Circulating Tumor Cell Analysis Approaches

Circulating tumor cells (CTCs) are cancer cells that have escaped from the primary tumor and are disseminated throughout the body via the blood circulation as part of the metastatic process [46,47]. CTCs present a unique opportunity to analyze rare/early metastatic events and metastatic progression by capturing and characterizing cells collected from patient blood samples [45,47]. CTCs can provide important insight into a patient’s individual disease and offer an opportunity for single or rare cell analyses in the form of a minimally-invasive, real-time “liquid-biopsy” for monitoring disease progression and treatment responses [48,49]. The presence of CTCs has been associated with poor prognosis and treatment responses in the clinical setting [50] and CTCs can also be used as predictive markers to increase treatment efficacy [51]. This could eventually reduce healthcare costs and be a valuable tool for personalized treatment [52,53,54].

**Figure 2:**
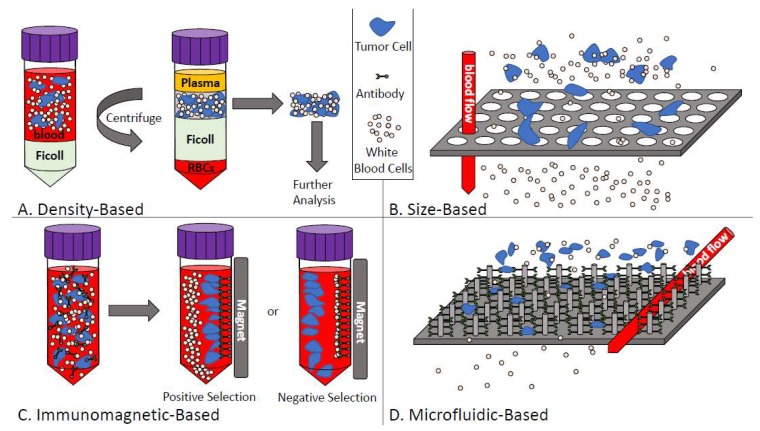
Overview of circulating tumor cell enrichment techniques. (**A**) Density-based CTC enrichment combines whole blood with density-gradient media such as Ficoll to separate based on cell density. Mononucleated cells including CTCs can then be recovered for further analysis; (**B**) Size-based CTC enrichment allows the smaller blood cells to pass through size restricted pores or channels while larger tumor cells are captured; (**C**) Immunomagnetic-based CTC enrichment either selects positively (for CTCs) or negatively (for white blood cells) using iron-labeled target antibodies; (**D**) Microfluidic-based CTC enrichment passes blood through either chip-based devices and/or antibody-coated micro-posts (depicted) to enrich CTCs from whole blood.

In metastatic cancer patients, the frequency of CTCs is approximately 1 CTC per 10^6^ leukocytes and, therefore, extremely sensitive and reproducible technologies are needed to analyze these cells in patient blood samples [45,47]. Because of the rare nature of CTCs, most assays use a combination of enrichment and detection/characterization techniques, which are described below and in Figure 2.

### 4.1. CTC Enrichment Techniques

#### 4.1.1. Density-Based Enrichment

Density-based isolation of CTCs takes advantage of the differences in density between CTCs (<1.077 g/mL) and blood cells (>1.077 g/mL) [55]. Ficoll-Paque^®^ (Sigma-Aldrich, St. Louis, MO, USA), OncoQuick^®^ (Greiner Bio-One, Monroe, NC, USA), and RosetteSep™ (StemCell Technologies, Vancouver, BC, USA) are all technologies that use density-based differences to separate CTCs from the blood. These approaches use density gradient medium to collect mononuclear cells (including CTCs) from blood samples (see Figure 2A) [56]. Similar to size-based enrichment techniques, collecting CTCs by density gradient allows for capture of both epithelial and mesenchymal CTCs and these techniques are also relatively easy and inexpensive to perform [55]. However, some shortcomings of density-based techniques include low specificity (because of the lack of specific selection for CTCs within the mononuclear fraction) combined with the opportunity for cross-contamination of CTCs with other cell types in the blood due to migration of cells to the plasma layer or due to the presence of clotting/aggregates [55].

#### 4.1.2. Size-Based Enrichment

Sized-based isolation of CTC capitalizes on the differences in size between CTCs (>8 µm) compared to leukocytes (<8 µm) [56,57]. Parsortix™ (Angle PLC; Surrey, UK), ScreenCell^®^ (Westford, MA, USA), and ISET^®^ (Rarecells, Paris, France) are examples of CTC technologies that use size-based techniques in conjunction with other methods to isolate the CTCs from blood samples. Typically, whole blood is passed through a filtration device with different pores or channels (usually 6–10 µm) or different filter-based approaches with the common goal of allowing isolation of CTCs from other blood components and retrieval of CTCs for further experimentation including multiplexed imaging, genetic analysis, or culturing (see Figure 2B) [56,57,58]. The advantages of size-based enrichment techniques include the straightforward and relatively inexpensive nature of these assays as well as the ability to identify both epithelial and mesenchymal CTCs [55]. However, size-based assays can be prone to clogging within the pores/channels and the low specificity of these assays sometimes causes small CTCs to be lost during the enrichment process [55].

#### 4.1.3. Immunomagnetic-Based Enrichment

The most widely used CTC enrichment/isolation technique is immunomagnetic-based selection of CTCs [58]. Immunomagnetic separation uses magnetic, bead-based separation technology [55]. CTCs are enriched from blood samples using antibodies conjugated to magnetic beads, which are designed to either positively select for CTCs by targeting various epithelial or tumor-specific antigens expressed by tumor cells or negatively select for CTCs by targeting contaminating blood cell antigens such as CD45 (see Figure 2C) [55,56]. AdnaTest (QIAGEN Hannover GmbH, Langenhagen, Germany), MACS^®^ (Miltenyi Biotec, Auburn, CA, USA), IsoFlux™ (Fluxion Biosciences, Alameda, CA, USA), and the CellSearch^®^ system (Menarini Silicon Biosystems Inc, San Diego, CA, USA) all use immunomagnetic approaches for CTC enrichment [55]. The various immunomagnetic technologies have different advantages. For example, MACS maintains cell integrity while AdnaTest uses defined markers and allows for downstream analysis of CTCs with the possibility of characterization of epithelial/mesenchymal phenotype. Through customizable IgG beads, Isoflux allows users to mix the antibodies of their choice when enumerating CTCs, which allows for very high customization. Lastly, the CellSearch^®^ is a regulatory-approved (United States Food and Drug Administration (FDA)), semi-automated technology that uses positive CTC enrichment via EpCAM (epithelial cell adhesion molecule) combined with image analysis. However, each of these technologies also has drawbacks including false positive/false negative isolation of CTCs (MACS^®^, AdnaTest) and limited flexibility in assay design or marker choice (AdnaTest, CellSearch^®^). In addition, the CellSearch^®^ only allows for capture of epithelial CTCs and has very limited capacity for downstream analysis of CTCs once they are identified. The greatest limitation with regards to immunomagnetic separation of CTCs from blood samples is the lack of a reliable “universal marker” that can be used independently of both the tumor type and the stage of disease progression [55].

#### 4.1.4. Microfluidic-Based Enrichment

To enrich for CTCs using microfluidic-based techniques, whole blood is passed through µm-sized channels by using chip-based devices designed with micro-channels etched or molded into surfaces such as glass, silicon, or polymers [56]. CTCs can then be captured by antibody-coated microposts or by size/deformability (see Figure 2D) [56]. Parsortix™, CTC-Chip/iChip, IsoFlux™, and GILUPI CellCollector™ (NanoMedizin, Potsdam, Germany) are all techniques that use microfluidics as a basis to enrich for and capture CTCs. Microfluidic-based techniques generally have high enrichment percentages and allow for the release of intact CTCs after enrichment to facilitate downstream analysis. However, similar to immunomagnetic enrichment techniques, these assays lack a “universal marker” targeting all cancer subtypes including cancers of advanced metastatic disease.

### 4.2. CTC Detection/Characterization Techniques

Once CTCs have been enriched from the blood, detection, enumeration, and/or molecular characterization can be carried out using a variety of protein-based and nucleic acid-based approaches, which are summarized in Table 1 [55].

#### 4.2.1. Protein-Based Detection and Characterization

Protein-based detection and characterization of CTCs can be carried out using different techniques such as slide-based immunofluorescence, flow cytometry, CellSearch^®^, and the CTC-Chip/iChip [57]. Typically this is done through the use of fluorescent-conjugated antibodies targeting epithelial antigens such as CK-19 and EpCAM on CTCs and identification through laser-based and/or image-based detection systems [56,57]. These approaches allow for analysis of large sample volumes with high specificity [55]. However, some image-based characterization techniques such as manual microscopy-based immunofluorescence analysis of stained CTCs can be very slow and labor-intensive while other methods such as flow cytometry have low sensitivity and can be technically challenging [55,56,57].

#### 4.2.2. Nucleic Acid-Based Detection and Characterization

Frequently used nucleic acid-based detection and characterization of CTCs include real-time (RT) and quantitative RT (qRT) polymerase chain reaction (PCR) to identify CTCs based on expression of specific genes of interest [57]. In addition, more sophisticated methods such as next-generation sequencing and genomic analysis (including at the single-cell level) are emerging as valuable (albeit technologically challenging) tools for detailed CTC characterization since it relates to disease characteristics. The high sensitivity of nucleic acid-based techniques allows for analysis of a small number of CTCs although the amplification bias of PCR can lead to false-positive results and/or low specificity of CTC detection [57]. In addition, these approaches cannot accurately quantify/enumerate the number of CTCs in a sample, don’t allow for visualization of CTCs, and do not allow for recovery and further analysis of CTCs [55].

### 4.3. Additional CTC Analysis Approaches

#### 4.3.1. Dielectrophoresis

Approaches such as DEPArray™ (Silicon Biosystems, Bologna, Italy) involve an electrophoretic-based cell-sorting and isolation platform for single-cell purification and analysis of live or fixed cells. DEPArray™ digitally sorts 100% pure subpopulations of cells from samples using a chip-based microfluidic cartridge and automated microscope image-based analysis [59,60]. This system does require that cells are already enriched from whole blood samples using one of the techniques described above [49]. Labeled cells are loaded into the DEPArray™ cartridge where electrodes are activated to form DEP cages by trapping the labeled cells. The cartridge is scanned in each desired fluorescence channel to identify target cells, which are moved into a designated area. Individual cells are then dispensed into a collection tube for further analysis with Silicon Biosystems CellBrowser™ software [59,60]. This allows for differential analysis and characterization of tumor cell populations using next-generation sequencing [61]. The DEPArray™ is best suited for small sample sizes (<10,000 cells) and is ideal for further molecular characterization of small pure CTC samples. With samples containing more than 10,000 cells, the assay may have reduced ability for single-cell purity/resolution [49,59,60].

#### 4.3.2. Direct Cellular Imaging

The Epic Sciences platform (San Diego, CA, USA) was designed for detection and molecular characterization of CTCs in whole blood regardless of the epithelial status and without an enrichment step [62]. To enumerate cells for protein biomarker analysis, the EPIC Sciences platform first consists of slide prep where whole blood is lysed and nucleated cells are deposited onto slides and frozen [62,63]. Slides are then immunofluorescently stained and scanned by using Epic’s rapid scanning method [62,63]. By assessing protein expression and morphology, Epic can differentiate between white blood cells and CTCs, which can then be characterized into different subsets including “traditional” CTCs (CK-positive), CTC clusters, CK-negative CTCs, and apoptotic CTCs or can be characterized by a custom-selected set of markers [62,63]. Studies have shown that the Epic Sciences platform has high cell recovery, high specificity, and high reproducibility for CTC detection and characterization [62].

Overall, there are a number of promising technologies being developed for tracking and characterizing metastasis and treatment response using CTCs. However, several challenges remain and further work is needed to understand the biology of CTCs in the context of metastatic progression in order to optimize their wide-spread use as clinical biomarkers. In order to do this, analysis of CTCs using in vivo preclinical animal models of metastasis provides an important opportunity to study CTCs and to develop and/or optimize CTC assays in controlled experimental metastasis studies. The remainder of this review will describe different approaches that can be used to study metastatic progression and CTCs in the preclinical setting, including details of each of the various available CTC assays and a summary of the current advances and challenges of preclinical CTC analysis.

## 5. Preclinical Models of Metastasis and CTC Generation

The presence of CTCs in the blood has been correlated with disease progression towards metastasis [45]. Therefore, in order to study CTCs in a preclinical setting, we must generate metastatic disease using in vivo animal models such as mice. There are many different preclinical mouse models of metastasis. Each has specific advantages and disadvantages, which largely differ in many facets including time to metastasis development, experimental costs, and patient specificity.

### 5.1. Spontaneous Metastasis Models

Preclinical in vivo models for spontaneous metastasis involve injection of cancer cells into their orthotopic site of origin [64,65]. For example, the correct orthotopic injection site for breast cancer cells is the mammary fat pad of female mice, while prostate cancer cells are injected into the prostate gland of male mice followed by monitoring of disease progression over time [64,65]. The advantage of spontaneous metastasis models is the ability to model and analyze all steps of disease progression including the growth of the primary tumor and eventual spontaneous metastasis to distant organs [64]. This allows for natural development and monitoring of cancer metastasis as it progresses in a biological setting. Therefore, this is a better approach to modelling the full spectrum of clinical disease compared to intravenous models (described below) [65]. However, natural disease progression in spontaneous metastasis models often takes a long time and primary tumor burden may become too great for the animal before metastasis can spontaneously occur. Therefore, depending on the aggressiveness of the orthotopically injected cells, there is no guarantee that metastasis will occur before the host is overcome by disease burden. To combat this, an alternative approach involves surgical removal of the primary tumor to allow for metastases to progress to a detectable size [66].

### 5.2. Experimental Metastasis Models

Experimental metastasis models involve injecting tumor cells directly into the blood circulation of the animal [66]. Using these models, tumor cells can be targeted for delivery to, and metastatic growth in, different organs depending on the route of the injection [64]. For example, tail vein injection targets cells towards the lung while the mesenteric vein targets cells towards the liver. Intra-cardiac injection is aimed, but is not precise, at targeting metastatic growth in the bone and/or brain [64]. Using this approach, tumor cells bypass the initial steps of primary tumor growth and intravasation, which reduces the time to metastasis and secondary tumor formation [64,66]. However, although injecting cells into the vasculature can result in a wide distribution of tumor cells throughout the body, the largest number of cells interacting with tissues will be located at the site of the first capillary bed encountered [66]. Other criticisms of the experimental metastasis model arise from the limited sensitivity of the assay based on the inability to precisely target the site of metastasis. [64]. Furthermore, bypassing the biological mechanisms of primary tumor growth and intravasation of cells may not fully represent metastasis in the clinical setting [66].

### 5.3. Genetically Engineered Mouse Metastasis Models

Genetically engineered mouse models (GEMMs) manipulate target oncogene or tumor suppressor expression in mice in order to promote tumor development [67]. Transgenic and knockout GEMMs have provided important models for identifying tumor-associated and metastasis-associated genes that can lead to tumor formation and disease progression [67,68]. However, since genetic mutations in GEMMs are simultaneously modified in all cells of the animal or in the targeted organ/tissue, these models do not accurately represent the natural progression of sporadic cancer events resulting from accumulated genetic events in single cells [67,68]. Cre-ERT, Cre-*loxP*, and Flp-*FRT*/Cre-*loxP* recombination systems have been applied in an attempt to mimic the accumulation of mutations in multistep carcinogenesis, but are still imperfect at emulating biological cancer progression [67,68]. Evaluating metastasis preclinically in GEMMs remains challenging. However, one approach that has been used to address this is orthotopic transplantation of GEMM-derived tumor fragments into secondary murine hosts, which has been shown to generate metastasis [67,68]. Similar to techniques used in spontaneous mouse models, implantation can be followed by surgical resection of the primary tumor to allow time for the development of metastatic disease. While there is much to be learned from GEMMs, these metastasis models remain time-consuming, laborious, and quite expensive [67].

### 5.4. Patient-Derived Xenograft Models of Metastasis

Patient-derived xenograft (PDX) models may circumvent many potential artifacts seen in other metastasis models. PDX mice are created by orthotopic or subcutaneous implantation of fresh human tumor samples into immunodeficient mice rather than using cultured cell lines [68]. The PDX model system is currently the only method that incorporates the inter-patient and intra-tumor heterogeneity that is present in human cancer by growing patients’ own tumor samples directly in mice [68]. This allows for a highly personalized study of tumor progression and treatment responses. Furthermore, PDX models have been shown to provide a continuous and renewable source of human CTCs [69]. A significant correlation has been shown between the presence of CTCs in early breast cancer patients and in mice. Therefore, these types of models may be used to evaluate in early metastatic events [69]. However, PDX models are not without limitations, which include high variability in engraftment rates based on tumor type and grade, time to metastasis progression, and the need for direct access to fresh clinical specimens for initial implantation [68]. Similar to other in vivo preclinical metastasis models involving injection of human cancer cells or tissues, the requirement for immunocompromised host mice reduces or eliminates the ability to study immune cell function and analyze immunotherapeutic strategies [68]. Lastly, similar to GEMMs, PDX mice are extremely time-consuming, labor-intensive, and costly.

## 6. Tracking Metastasis and CTCs in Preclinical Models

### 6.1. Technologies for Preclinical Evaluation of CTCs

**Figure 3:**
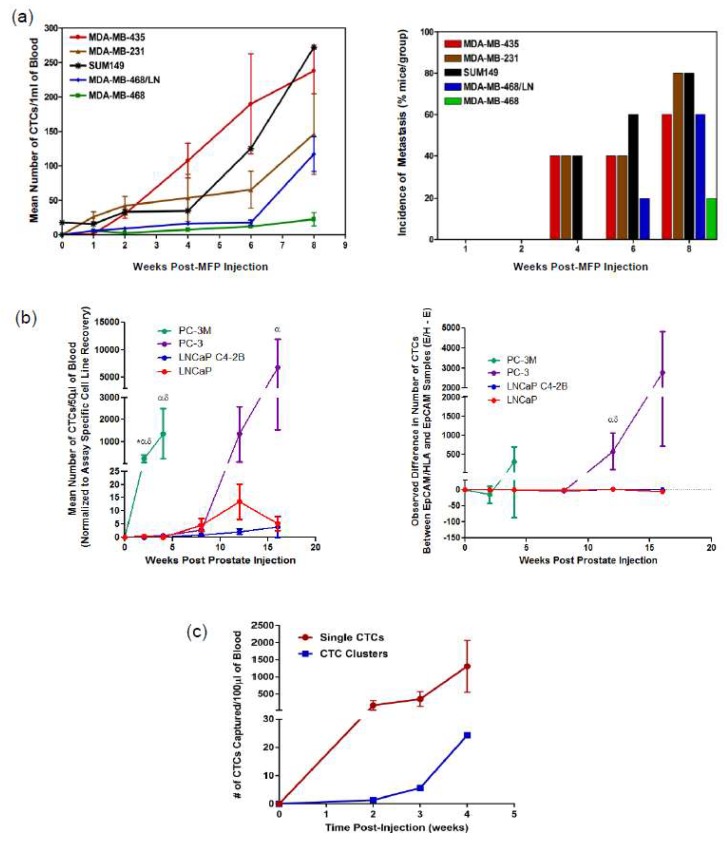
Technologies for CTC analysis and metastasis tracking in animal models of cancer. (**a**) Human breast cancer cell lines of differing metastatic abilities were injected into female nude (nu/nu) mice or NOD/SCID mice via the mammary fat pad (MFP). At several time points post-injection, mice were sacrificed and blood (1 mL) and tissues were collected and analyzed. CTC kinetics in blood was measured by flow cytometry (**left panel**; mean ± SEM, *n* = 5 mice/group) and the incidence of lung metastasis (% of mice in the group) was measured as assessed histopathology (**right panel**). Adapted from Reference [72]. (**b**) Human prostate cancer cells of differing metastatic ability were injected into male nude mice via the right dorsolateral lobe of the prostate gland. At several timepoints post injection, mice were sacrificed and blood (100 µL) was collected and split into two 50 µL aliquots before analysis using an adapted CellSearch^®^ CTC assay to assess CTC kinetics between epithelial (LNCaP, C4-2B) versus mesenchymal (PC-3M, PC-3) cell lines (**left panel**, mean ± SEM, *n* = 5–12 mice/group). To compare the difference in CTC number detected using EMT-dependent (EpCAM+) versus EMT semi-independent (EpCAM+/HLA+) adapted CellSearch^®^ assays in matched samples (**right panel**; mean ± SEM); positive values = more CTCs detected with EMT semi-independent assay, negative values = more CTCs detected with EMT-dependent assay. * = significant difference relative to PC-3; α = significant difference relative to LNCaP C4-2B; δ = significant difference relative to LNCaP (*p* ≤ 0.05). Adapted from Reference [45]. (**c**) Human PC-3 prostate cancer cells (mesenchymal phenotype) were injected into male nude mice via the right dorsolateral lobe of the prostate gland. At several timepoints, post injection blood (100 µL) was serially collected and analyzed using the Parsortix™ CTC analysis platform.

In the preclinical setting, multiple CTC enumeration technologies are becoming available for use in tracking metastasis, developing biomarkers, and assessing molecular analysis of CTCs. These include a number of new emerging technologies that have been shown to process the small volumes of blood obtained from preclinical models such as the VTX-1 platform (Vortex Biosciences, Menlo Park, CA, USA) [70] and the ApoStream technology (ApoCell, Houston, TX, USA) [71]. Our research group has demonstrated the value of using three different technologies for CTC analysis and metastasis tracking in animal models of cancer including flow cytometry, CellSearch^®^, and Parsortix™ (see Figure 3) and this section will focus on these technologies. Each approach is described below and the advantages and disadvantages are discussed and summarized in Table 2.

#### 6.1.1. Flow Cytometry

Flow cytometry can be used for characterizing tumor cell dissemination patterns and kinetics in preclinical models of cancer metastasis [64]. Using flow cytometry in a preclinical setting, human cancer cells can be effectively identified among mouse leukocytes. This process has been described previously by Allan et al. (2005) [73]. Briefly, flow cytometry can be used to identify CTCs based on their size, positive staining with a FITC-conjugated anti-human leukocyte antigen (HLA) antibody, negative staining with a phycoerythrin (PE)-conjugated anti-mouse pan-leukocyte CD45 antibody, and aneuploidy DNA content based propidium iodide (PI) staining [64,73].

This CTC analysis approach was one of the first to allow for early detection and quantification of rare metastatic tumor cells in the blood of mice and this led to evidence of CTC contribution to the development of metastatic disease [64]. Utilizing the preclinical potential of flow cytometry analysis of CTCs, studies have assessed tumor cell dissemination patterns using spontaneous and experimental CTC/metastasis models [64] and tracked the kinetics of CTC generation in vivo by analyzing multiple breast cancer cell lines and their relationship between CTCs and metastasis (see Figure 3a) [72].

Flow cytometry is one of the most cost-effective CTC enumeration technologies available for preclinical CTC analysis, which allows for harvesting of viable cells for downstream analysis if a fluorescence activated cell sorting (FACS) instrument is used. However, this particular method of CTC enumeration relies heavily on expression of HLA on the human tumor cells [73]. As such, the use of cell lines with lower or absent levels of HLA expression may present difficulties with this technique. This is particularly relevant when considering future application of this assay to preclinical models that make use of primary patient-derived tumor cells or CTCs. These CTCs are likely to be much more heterogeneous than immortalized cell lines not only in terms of expression of HLA or other cell surface markers but also in terms of heterogeneous size and enhanced fragility [74]. It is also important to note that the lower detection limit for sensitivity for this flow cytometry assay is 1 human CTC in approximately 10,000 mouse leukocytes [73]. The addition of a negative immuno-magnetic depletion step targeting CD45+ cells before flow cytometry analysis increases the lower detection limit to 1 CTC in 100,000 mouse leukocytes [73], which is improved but still 10-fold lower than the clinical conditions of 1 CTC per 10^6^ leukocytes [73]. The largest disadvantage of using this particular HLA-based flow cytometry CTC enumeration assay is the inability to translate into a clinical setting, since this technique exploits the differences in properties between mouse cells and human cells to enumerate CTCs, which would not be present in patients.

#### 6.1.2. CellSearch^®^

The CellSearch^®^ system is the only FDA-approved technology for enumeration of CTCs in metastatic breast, prostate, and colorectal cancer patients. Therefore, it is considered the clinical “gold-standard” in CTC enumeration technology [74]. It provides a standardized method for highly sensitive detection and quantification of CTCs from human blood samples. The CellSearch^®^ first automates blood sample preparation using the CellTracks AutoPrep^®^ system and then scans the samples using the CellTracks Analzyer II^®^. CTCs are enriched using immunomagnetic antibodies for EpCAM, which is an epithelial cell adhesion molecule present on CTCs but not on leukocytes. To identify CTCs, cells are stained for cytokeratins (CK) 8, 18, and 19 (epithelial filament proteins also expressed by CTCs but not leukocytes), anti-CD45, a leukocyte marker, and DAPI to identify the nucleated cells. After automated scanning of the blood sample, all potential CTCs are presented to the user who must select, via semi-quantitative analysis, which events are true CTCs. The system allows for (but does not necessitate) an additional user-defined marker of interest using a fluorescein isothiocyanate (FITC) fluorescence channel to allow for single cell characterization of CTCs [74].

**Figure 4:**
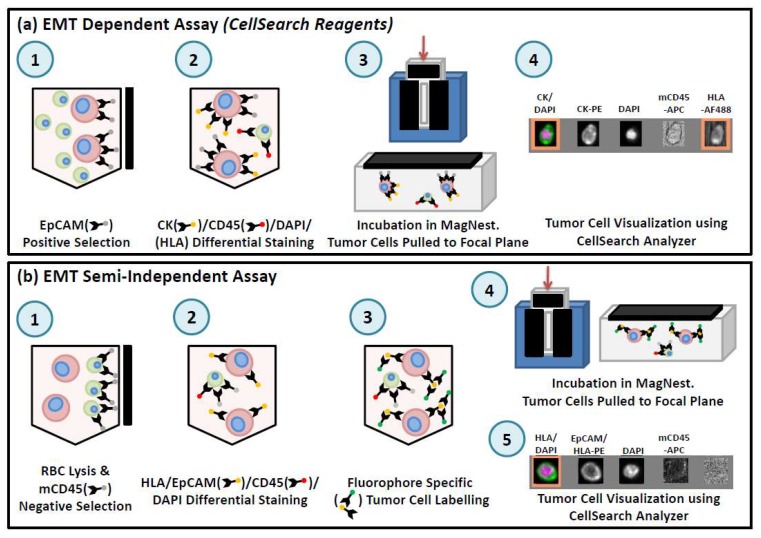
Overview of workflow for preclinical CTC assays using CellSearch^®^. (**a**) For the EMT-dependent CTC assay, 50 µL of whole mouse blood is incubated with components of the CellSearch^®^ CTC kit (anti-EpCAM ferrofluid, Capture Enhancement Reagent, Nucleic Acid Dye, Staining Reagent, Permeabilization Reagent) as well as anti-mouse CD45-APC and anti-human HLA-AlexaFluor488. Samples are manually immuno-magnetically separated and transferred to a MagNest™ for analysis using the CellSearch^®^. EpCAM+/CK+/DAPI+/CD45−/HLA+ cells with a round/oval morphology were classified as CTCs. **(b)** For the EMT semi-independent CTC assay, 50 µL of blood is lysed with NH_4_Cl. Samples are washed and labeled with anti-human HLA-PE, anti-human EpCAM-PE, and anti-mouse CD45-APC. Samples are washed and manually immunomagnetically enriched using the EasySep APC Positive Selection kit (StemCell Technologies, Vancouver, BC, Canada), incubated with CellSearch^®^ Permeabilization Reagent and Nucleic Acid Dye and transferred to a MagNest™ for analysis using the CellSearch^®^. EpCAM/HLA+/DAPI+/CD45− cells with a round/oval morphology were classified as CTCs.

In order to use CellSearch^®^ for preclinical analysis of CTCs in blood from mouse models, it is necessary to adapt the human CTC assay for use with small blood volumes (ranging from 50 µL for serial blood draws to 1 mL for terminal blood draws) [45,74] (see Figure 4). This adapted assay can capture CTCs based on the exact criteria of the human test (EpCAM+/CK+/CD45−) (see Figure 4a) or by including HLA as a positive selection marker to capitalize on the human-in-mouse model and to broaden CTC capture criteria to include non-epithelial CTCs (see Figure 4b). Multiple components of the CellSearch^®^ kit can be used to achieve this in combination with manual immunomagnetic separation of the sample. The enriched sample is then transferred to a MagNest™ for analysis using the CellTracks Analyzer II^®^ [45,74].

Preclinical studies using the adapted CellSearch^®^ assay have illustrated the important impact of the epithelial-to-mesenchymal transition (EMT) as it relates to CTC generation, CTC detection, and metastatic progression. Lowes et al. (2016) [45] observed that mesenchymal prostate cancer tumors shed greater numbers of CTCs more quickly and had greater metastatic capacity compared to tumors with an epithelial phenotype. In addition, the traditional EpCAM-based CellSearch^®^ assay missed significantly more mesenchymal prostate cancer CTCs when compared to the adapted HLA-based CTC enumeration assay [45,74]. Notably, although the EpCAM-based CellSearch^®^ assay was able to capture the majority of CTCs shed in early-stage disease with development of mesenchymal characteristics in later-stage disease, the CellSearch^®^ assay became more inefficient at enumerating the most aggressive mesenchymal CTCs (see Figure 3b) [45].

The highly standardized nature of CellSearch^®^ provides the advantages of reliability, consistency, and high sensitivity when used in the setting of preclinical CTC analysis. Furthermore, because the CellSearch^®^ is approved for use in the clinical setting, preclinical CTC studies using the CellSearch^®^ can be readily translated to the clinic. However, the CellSearch^®^ only provides one additional fluorescence channel for added characterization of CTCs and is, therefore, limited with regards to user customization and downstream molecular characterization [74]. CellSearch^®^ is also very expensive both in terms of initial infrastructure investment as well as assay reagents, which creates a barrier for researchers with limited funds. However, the largest obstacle when using the CellSearch^®^ is the dependency on the epithelial marker EpCAM to enumerate the CTCs from blood samples.

#### 6.1.3. Parsortix™

The Parsortix™ system uses a combination of microfluidic and size/deformability-based approaches to separate CTCs from blood samples [75]. The cassettes use a step-wise system, which gradually narrows to either 6.5, 8, or 10 µm depending on which cassette the researcher chooses. Since CTCs are generally larger than blood cells, the stepwise microfluidic system allows for the flow of blood cells through the cassette while the CTCs are captured. Different cassette sizes allow the researcher to select a balance between high specificity/purity or high sensitivity of captured cells [76]. Once the CTCs are enumerated from the blood, the Parsortix™ system allows the user a variety of downstream options. These include harvesting the collected CTCs, using back-flow to gather the cells for further molecular or functional analysis, or staining the CTCs directly within the cassette (workflow described on the Parsortix™ website [77]). Studies have shown in cell spiking experiments that subsequent harvest of captured CTCs ranges from 54% to 69% of total captured CTCs with 99% viability of harvested cells [76]. To enumerate captured CTCs, researchers can choose to fix the cells with or without permeabilization of the cell membrane and can stain with custom-selected fluorescent antibodies to allow for visualization of the cells and quantitative analysis.

Analysis of CTC generation and dissemination in animal models of prostate cancer demonstrates the value of using Parsortix™ for CTC analysis in the preclinical setting. In particular, Parsortix™ is able to detect and track the kinetics of highly mesenchymal CTCs generated following orthotopic injection of the aggressive PC-3 human prostate cancer cell line (see Figure 3c). In addition, Parsortix™ allows for the analysis of both single CTCs and CTC clusters, the latter of which are difficult to assess using flow cytometry or CellSearch^®^ but have been shown to be biologically important for disease progression [78]. This suggests that the size/deformability-based, epithelial-independent Parsortix™ may be a better tool for analysis of CTCs throughout EMT and disease progression to metastasis.

Parsortix™ is less expensive compared to the CellSearch^®^ and allows for full customization of subsequent staining of captured CTCs. It also allows the user to harvest captured CTCs for downstream functional and molecular analysis, which is advantageous for preclinical biomarker development and metastasis studies. Compounded by the ability to analyze <100 µL of blood compared to the necessary 7.5 mL of blood using the CellSearch^®^, Parsortix™ is much more suited for preclinical analysis of CTCs in mice [76]. Parsortix™ is approved for use in the clinical setting in Europe through the CE Mark system, which suggests that preclinical CTC studies using Parsortix™ could be readily translated to the clinic. However, the main advantage of using Parsortix™ over CellSearch^®^ is the ability to enumerate CTCs independent of their epithelial or mesenchymal status [76,79]. This allows for preclinical studies comparing the capture of mesenchymal CTCs throughout disease progression, which in turn could lend insight as to which patients might be at the most risk of false-negative CTC analysis using the CellSearch^®^ in the clinical setting. However, as previously mentioned, it has been shown that depending on which size cassette the researcher chooses, recovery of CTCs from blood samples varies significantly, which may be problematic for analysis of rare CTC populations [76]. It has also been suggested that high contamination of leukocytes within captured CTC samples may provide difficulties with molecular analysis of harvested cells [79].

### 6.2. Elucidating the Biology of CTCs and Metastasis via Preclinical Models

Overall, the studies described above provide useful technical guidelines for researchers to use while planning preclinical metastasis studies and selecting the CTC technology that will best suit their needs. While the described studies have provided an improved understanding of the biology of CTCs and their relationship with metastatic disease, several other elegant studies (described below) have also contributed valuable knowledge in this area.

A preclinical study conducted by Baccelli et al. (2013) [80] demonstrated that CTCs isolated from breast cancer patient samples contained transplantable metastasis-initiating cells, which gave rise to metastatic disease in mice. Co-expression of CD44v6 and MET was observed to be particularly important for metastasis initiation where interaction with HGF in breast cancer metastases enhanced MET-kinase signaling [80]. Another study by Vishnoi and colleagues (2015) [81] used patient samples to culture CTC-derived 3D tumorspheres that allowed for the assessment of biomarker profiling and biological characteristics. Using multiparametric flow cytometry, they revealed that enriched CTC populations from breast tumors had unique gene signatures. They also observed that FACS-sorted CTC populations, which expanded into 3D CTC tumorspheres in non-adherent stem cell conditions, had suggestive metastatic competency and had cellular protrusions. Through their findings, they elucidated mechanisms for the generation of tumor-associated vesicles (oncosomes) and their related role in mediating intracellular signaling. Lastly, they were able to characterize the 3D CTC tumorspheres as non-hematopoietic, tumorigenic, and as containing stem-cell properties [81]. In a report from Zhang et al. (2013) [82], CTCs isolated from patients with breast cancer were characterized and used to create multiple cell lines for subsequent in vivo and in vitro work. This study identified a potential signature for breast cancer brain metastasis and analyzed these cells for invasiveness and metastatic competency. With the development of brain metastatic breast cancer CTC cell lines, this group is now exploring potential protein signatures and mechanisms of brain cancer metastasis in the preclinical setting [82]. Lastly, a review by Kang and Pantel (2013) [83] nicely summarizes how animal models of CTCs mimic the complexity of patient cancers in the clinic and how preclinical studies have identified many important mechanisms of metastasis including pathways, inhibitors, and gene signatures that are accelerating the identification and implementation of clinically relevant CTC information.

## 7. Conclusions and Future Directions

In summary, preclinical CTC analysis has provided insight into mechanisms of cancer metastasis, into the transition between epithelial-to-mesenchymal phenotypes, and into potential new biomarkers. Technological advances in single-cell analysis have also elucidated potential gene expression profiles and cell mutations, which influence cell aggressiveness. Future work focused on CTCs will allow researchers to continue to better understand the biology underlying the metastatic process and allows for metastasis-specific drug development as well as assessment of therapy response. Preclinical studies will continue to identify potential biomarkers, gene signatures, survival mechanisms, and novel mechanisms of metastasis through CTC analysis. Single-cell genomic analysis of CTCs will provide more insights into phenotypic changes, which may lead to disease progression and metastasis. By converging preclinical and clinical CTC studies, there has already been great development in the intellectual framework of the metastasis field, which has generated many unresolved questions to be addressed in future studies to improve cancer therapies [82]. The accessibility of liquid biopsies such as CTC assays will allow for continued focus on combined preclinical/clinical work by elucidating mechanisms of cancer progression in patients’ own CTC samples. This targeted work will not only benefit patients with metastatic cancer, but also patients with early-stage cancers who may be at high risk for cancer progression.

## Figures and Tables

**Table 1:** Comparison of protein-based and nucleic acid-based approaches for circulating tumor cell characterization in patient samples.

Type	Approach	Sample Volume Requirement *	Ability for CTC Quantification	High Specificity	High Sensitivity	Labor-Intensive/Challenging	Down-Stream Analysis
**Protein-Based**	Immunofluorescence	Small	Yes	Yes	No	Yes	Yes
	Flow Cytometry	Medium	Yes	Yes	No	Yes	Yes
	CellSearch^®^	Large	Yes	Yes	Yes	No	No
	CTC-Chip/iChip	Large	Yes	Yes	Yes	Yes	Yes
	AdnaTest	Large	No	Yes	Yes	No	No
	Isoflux	Large	Yes	Yes	Yes	No	Yes
**Nucleic Acid-Based**	RT-PCR	Small	No	No	Yes	No	No
	qRT-PCR	Small	No	No	Yes	No	No
	Next-Gen Sequencing	Small	No	Yes	Yes	Yes	Yes

* Small: ≥100 µL, Medium: ≥1 mL, Large: ≥7 mL.

**Table 2:** Comparison of preclinical CTC analysis approaches.

Feature	Flow Cytometry	CellSearch^®^	Parsortix™
Sensitivity	**☑**	**☑☑☑**	**☑☑☑**
Captures cells independent of phenotype	**☒**	**☒**	**☑**
Ability for downstream analysis	**☑**	**☒**	**☑☑☑**
Accurate low blood volume analysis	**☒**	**☑**	**☑☑☑**
Ease of process	**☒**	**☒**	**☑**
Low cost	**☑☑☑**	**☒**	**☑☑**
Support for research/flexibility	**☑☑**	**☒**	**☑☑☑**
Clinical relevance	**☒**	☑☑ (FDA)	☑ (CE Mark)

☒ = disadvantages; ☑ = advantages.
